# The use of collagen matrix (Ologen) as a patch graft in glaucoma tube shunt surgery, a retrospective chart review

**DOI:** 10.12688/f1000research.9232.1

**Published:** 2016-08-01

**Authors:** John D. Stephens, Steven R. Sarkisian, Jr.

**Affiliations:** 1 Dean McGee Eye Institute, University of Oklahoma School of Medicine, Oklahoma City, OK, 73126-0901, USA

**Keywords:** Ophthalmology, glaucoma, glaucoma surgery, glaucoma tube shunt, glaucoma patch graft, collagen matrix

## Abstract

**Purpose: **To determine the safety and efficacy of collagen matrix as a patch graft in glaucoma drainage surgery. Collagen matrix grafts may be advantageous because they do not need to be harvested from human donors.

**Methods:** An institutional, retrospective review of 43 patients with at least 12 months follow-up status post-glaucoma drainage implant surgery were evaluated for signs of tube erosion after initial placement of collagen matrix patch graft.

**Results:** Forty-one of 43 eyes (95.3%) required no intervention for patch graft melting with tube erosion. Average time of follow-up was 32 months (range: 12-45). Two cases had tube erosion at 4 months and 26 months post-op requiring tube revision, which was successfully revised with conjunctiva (4 month erosion) and donor sclera (26 month erosion).

**Conclusion:**  Our results suggest that collagen matrix patch grafts may be used successfully as a patch graft in glaucoma tube shunt surgery, and may be advantageous because they do not have to be harvested from human donors. It is possible that exposure rates may be higher after longer follow-up and with larger numbers of patients. Further research is needed to compare Ologen to traditional graft materials to conclusively determine the safety and efficacy of collagen matrix as a novel patch graft material.

## Introduction

The use of glaucoma drainage implants to treat difficult glaucoma cases has increased in the past two decades ^1^. These devices drain aqueous through a silicone tube to a reservoir plate covered by Tenon’s capsule and conjunctiva. The tube is then covered by one of several materials to prevent exposure to the overlying conjunctiva. Although most complications are transient and self-limited, glaucoma drainage procedures carry the risk of persistent corneal edema, tube erosion, endophthalmitis/blebitis, and tube migration, among other complications ^2^. Tube shunts in particular carry the risk of patch graft thinning and exposure of the subconjunctival portion of the shunt tube, which is a risk factor for infectious endophthalmitis ^3, 4^. Prompt identification and revision of exposed patch grafts with collagenous human autograft or allograft material is therefore recommended ^5^.

**Figure 1.**
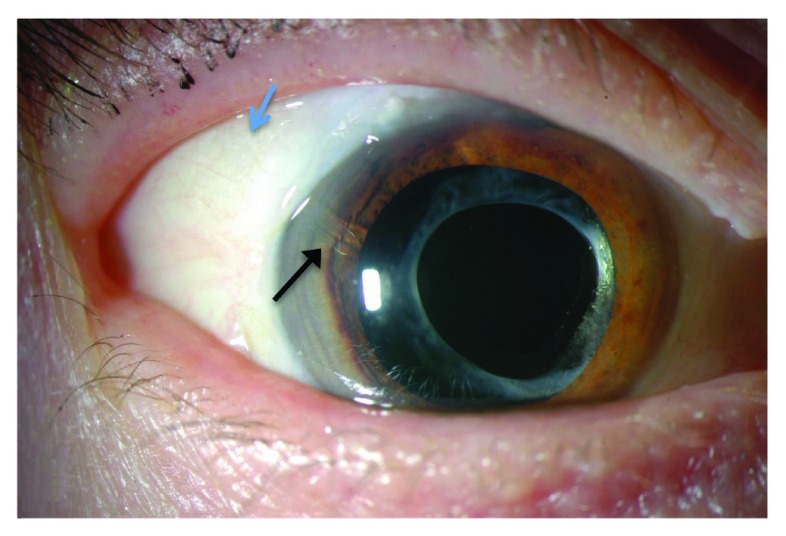
Slit lamp photo demonstrating cosmesis of Ologen patch graft. Black arrow: tube in anterior chamber. Blue arrow: Ologen patch graft.

Several patch graft materials have been used. These include pericardium, fascia lata, cornea, sclera, and amniotic membrane ^6, 7^. Ologen (Aeon Astron Europe BV, Leiden, the Netherlands) is a porcine-derived biodegradable collagen matrix implant which has been studied and used as an adjunct to trabeculectomy ^8, 9^. A recent case report showed successful use of Ologen as a patch before closing the conjunctiva in a case of tube erosion ^10^. To our knowledge, Ologen has not been used as a primary patch graft in glaucoma tube shunt procedures. Collagen matrix may be advantageous because it does not need to be harvested from human donors and is less expensive than other patch graft materials. This is particularly important considering that Medicare (the federal health insurance program for people who are 65 or older, medicare.gov) now no longer reimburses for any patch graft material when combined with a tube shunt procedure (former CPT code 67255). Additionally, Ologen appears clear under the conjunctiva and provides improved cosmesis compared to other patch grafts ( Figure 1, printed with permission courtesy of Steven R. Sarkisian, jr.). The purpose of this study was to determine the safety and efficacy of collagen matrix as a patch graft in glaucoma tube shunt surgery.

## Materials and methods

This study was approved and monitored by the Institutional Review Board at the University of Oklahoma Health Science Center (IRB# 3425; reference #652312). Permission to publish clinical details and images was obtained for each subject. Potential subjects were identified by reviewing case logs of a single attending surgeon (S.R.S.). Charts of consecutive patients undergoing glaucoma tube shunt surgery with placement of collagen matrix patch graft between July 2009 and December 2010 were reviewed. Charts were excluded if the patient had less than 12 months of follow-up data. Forty-three eyes of 40 patients were identified. Demographic and clinical information of the patients is listed in Table 1. The primary outcome measure of this study was post-operative tube exposure requiring revision.

**Table 1.** Demographic and Clinical Information.

Age (+/- SD)	63 (+/- 20)
Gender Male Female	20 23
Ethnicity Caucasian African American Native American Hispanic Not identified	23 7 7 1 5
Diagnosis Primary open angle glaucoma (POAG) Non-POAG	28 15
Quadrant Superotemporal Inferonasal	40 3
Type of tube shunt Ahmed Barveldt	37 6
Tube location Anterior chamber Sulcus Pars plana	37 5 1
Average months of followup	32 (range 12–45)

## Surgical technique

**Figure 2.**
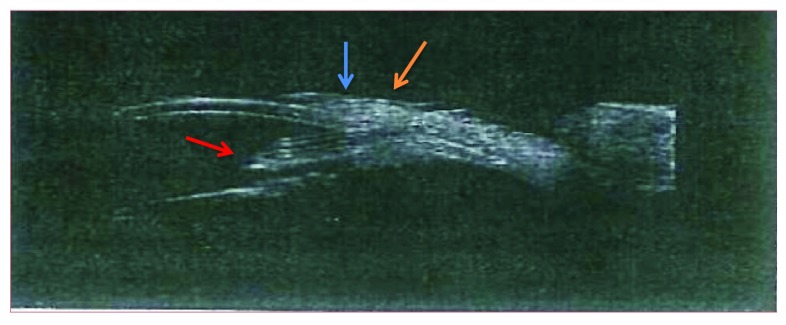
Ultrasound biomicroscopy taken three years post-operatively of tube shunt with Ologen patch graft. Red arrow: tube in anterior chamber. Blue arrow: conjunctiva over patch graft. Orange arrow: Ologen patch graft.

The glaucoma drainage implant of choice was placed in the usual fashion ^11^. Once the tube was secured to the sclera, the collagen patch graft was used to cover the tube ( Figure 2). The Ologen to cover a tube comes as a 10×10×2 mm sheet. Presoaking the collagen is not necessary and is, in fact discouraged because once wet, the collagen becomes difficult to cut and can tear easily. While dry, the collagen sheet was cut to size to cover the tube per the surgeon's preference. Although some surgeons may desire to suture the collagen in place, we find this unnecessary as the collagen quickly picks up moisture from the scleral bed, does not slide out of place easily and never moves post-operatively once the conjunctiva is closed. However, great care is taken to ensure that the collagen is fully covered and the conjunctiva covering it is not under tension. Every effort must be made to be certain there is no chance that any part of the collagen is exposed and the conjunctiva is well secured. Once the conjunctiva was closed, a small amount of saline was placed in the anterior chamber and a fluorescein strip was used to verify the absence of leakage.

## Results

A brief summary of results is displayed in table 2. Forty-one of 43 (95.4%) eyes with Ologen patch graft required no intervention for patch graft melting with tube erosion. The average time of follow-up was 32 months (range 12–45 months). Two cases had tube erosion requiring revision. These occurred at 4 months and 26 months post-operatively. The first patient was an 86-year-old Caucasian woman with open angle glaucoma and a history of iritis. She had partial exposure of the patch graft after 1 week and full exposure at 4 months. She underwent successful tube revision with conjunctiva for a total follow up of 32 months. The second erosion occurred in a 74-year-old Caucasian woman with open angle glaucoma and long-standing diabetes mellitus. The erosion occurred at 26 months and was successfully repaired with donor sclera for a total follow up of 32 months. Neither patient developed signs of endophthalmitis during their clinical course. Both of these patients had Ahmed valves placed in the superotemporal quadrant. One patient in this study, a 63-year-old man with open angle glaucoma, developed partial tube exposure on post-operative day 10 but did not require revision. He underwent placement of Baerveldt shunt in the inferonasal quadrant.

**Table 2.** Summary of results.

Number of eyes	43
Tube erosions (%)	2 (4.7%)
Average time to erosion	15 months (2 months, 36 months)
Successful revision	2/2 (100%)
Average time of follow up	32 months (range 12–45 months)

The use of collagen matrix (Ologen) as a patch graft in glaucoma tube shunt surgery, a retrospective chart review data spreadsheet Collagen matrix patch graft data are provided in a spreadsheet. Description of the dataset is provided in the text file. Click here for additional data file. Copyright: © 2016 Stephens JD and Sarkisian, Jr. SR 2016 Data associated with the article are available under the terms of the Creative Commons Zero "No rights reserved" data waiver (CC0 1.0 Public domain dedication).

## Discussion

To our knowledge, no study has investigated the use of collagen matrix material as a primary patch graft in glaucoma tube shunt surgery. Previous studies have reported rates of patch graft erosion. Gedde *et al*. reported tube erosion in five of 107 eyes (4.6%) in the tube versus trabeculectomy study at 5 years of follow-up ^11^. In a study of 702 patients, Levinson *et al.* reported an exposure rate of 5.8% at a mean follow up of 36 months ^12^. Additionally, Muir *et al.* reported an exposure rate of 6.2% in 1073 patients followed for an average of 41 months ^13^. The erosion rate in our study, 4.7%, is comparable to these previous studies.

Several factors may predispose patients to patch graft erosion. In a cohort study of 121 eyes, Koval *et al.* identified Hispanic ethnicity, neovascular glaucoma, previous trabeculectomy, and combined surgery as potential risk factors for tube shunt exposure ^14^. In the aforementioned study by Muir *et al*., female gender and white race were associated with an increased risk of graft exposure. Uveitis, diabetes, and type of tube shunt were not associated with increased risk ^13^. Mechanical and immunologic factors may also contribute to graft erosion ^15^. Both of the patients with graft erosion in our study had histories suggestive of poor wound healing and/or ocular inflammation. One had long-standing diabetes mellitus without a diagnosis of neovascular glaucoma. The second patient with erosion in our study had a history of iritis.

Ologen encapsulates when not exposed to aqueous and does not biodegrade. It is possible that the patch graft erosions in our study occurred because the Ologen was exposed and not well-covered initially, leading to patch melting. Care must be taken to not use Ologen if the conjunctiva is under tension when it is closed.

There are several limitations to this study. First, given its relatively small sample size and limited duration, further studies are necessary to determine the safety and efficacy of Ologen collagen matrix patch grafts compared to other commonly used materials. There are inherent limitations in a retrospective chart review, including lack of randomization of patients, lack of comparative control group and incomplete follow-up by patients not reviewed for this study. A prospective, large, controlled study is needed to compare erosion rates of Ologen to other graft materials. It is possible that collagen matrix patch grafts may be used successfully in glaucoma tube shunt surgery. They may be advantageous because they do not need to be harvested from human donors, are less expensive, and provide improved cosmesis compared to other commonly used materials. Further study is required to evaluate the long-term use of Ologen as a patch graft.

## Data availability

The data referenced by this article are under copyright with the following copyright statement: Copyright: © 2016 Stephens JD and Sarkisian, Jr. SR

Data associated with the article are available under the terms of the Creative Commons Zero "No rights reserved" data waiver (CC0 1.0 Public domain dedication). 

F1000Research: Dataset 1. The use of collagen matrix (Ologen) as a patch graft in glaucoma tube shunt surgery, a retrospective chart review data spreadsheet. 10.5256/f1000research.9232.d130894 ^16^

## Ethical considerations

This study was approved and monitored by the Institutional Review Board at the University of Oklahoma Health Science Center (IRB# 3425; reference #652312). Permission to publish clinical details and images was obtained for each subject.
