# Supplementary material for: The use of collagen matrix (Ologen) as a patch graft in glaucoma tube shunt surgery, a retrospective chart review
Source: F1000Res. 2016 Aug 1;5:1898. [Version 1] doi: 10.12688/f1000research.9232.1 (PMC5288683; doi:10.12688/f1000research.9232.1)
Supplement: Peer review history

# Peer review: Referee response for version 1

**DOI:** 10.5256/f1000research.9936.r19745

**Published:** 2017-02-01

**Authors:** Nathan M. Radcliffe

**Affiliations:** 1 Department of Ophthalmology, School of Medicine, New York University (NYU), New York, NY, USA

The authors provide a useful report on the practice of replacing a human allograft cornea, sclera, or pericardial graft with porcine collagen matrix (Ologen) to cover the tube entry site during glaucoma drainage device placement. The authors give a reasonable rational for the use of collagen, namely cosmesis, cost and the ability to avoid transplanted human tissue. It should be noted that porcine material is not for everyone, and the use of this material should be discussed with patients prior to placement.  The strengths of the study are a reasonable follow up duration and a sufficient number of patients, given that this is the first report of this surgical modification. I say that this number is reasonable, because after 40 cases of a new technique, it is appropriate for the surgeon to pause, carefully analyze the experience, and report the results to the scientific community.

 The limitations of the study include its retrospective nature and the lack of a control arm. A greater number of patients would include the power of the study, and any future reports on this topic would do well to include more patients. The authors provide adequate support from the literature that a roughly 5% tube erosion rate, as was seen in this study, is the standard.

 Another limitation of this study relates to the fact that there is a growing body of evidence that glaucoma tube shunt surgery may be safely performed without the use of any patch graft material.  Using a long scleral tunnel needle technique, Oscar Albis and colleagues ^1^ reported a 0% erosion rate in 106 Mexican children followed after Ahmed valve placement with no patch graft.  The mean follow up was just over two years, no patients were followed for less than six months and some were followed for up to eight years.  While this data is encouraging, the reality of the situation is that in the United States, many surgeons are still using patch graft materials, and these materials do have problems, cosmesis and cost being among them.  I applaud the authors for making a valuable contribution to the surgical literature and for answering a straightforward but important question: is it reasonable to substitute collagen matrix for other graft materials in glaucoma drainage device surgery?  Yes.

I have read this submission. I believe that I have an appropriate level of expertise to confirm that it is of an acceptable scientific standard.

## References

[1] Albis-DonadoOGil-CarrascoFRomero-QuijadaRThomasR: Evaluation of Ahmed glaucoma valve implantation through a needle-generated scleral tunnel in Mexican children with glaucoma. *Indian J Ophthalmol*.58(5) : 10.4103/0301-4738.67039 365-73 10.4103/0301-4738.67039 20689189PMC2992909

# Peer review: Referee response for version 1

**DOI:** 10.5256/f1000research.9936.r15561

**Published:** 2016-08-09

**Authors:** Arsham Sheybani, Bliss O’Bryhim, Ankur Sudhir Gupta

**Affiliations:** 1 Department of Ophthalmology and Visual Sciences, School of Medicine, Washington University in St. Louis, St. Louis, MO, USA

“The Use of Collagen Matrix As a Patch Graft in Glaucoma” is a well designed study on the use of Ologen as a primary graft in glaucoma shunt surgery. The title is appropriate, though it may benefit from the inclusion of the word “primary” (...”as a primary patch graft”) to distinguish it from other publications on the use of Ologen as a patch graft. The method and analysis are well explained.

 Although not the primary intent of the study, additional statistical analysis would be useful. This would include reporting the mean change in IOP and medication use post-operatively. While we do not expect that grafts would affect IOP or medication use, data showing IOP reductions could be useful in drawing this conclusion. Reporting the number of previous surgeries on the study eyes and eyelids may elucidate root causes for complications. At present, the supplemental data included only describes surgeries and procedures that occurred after the initial tube placement.

 The conclusions are sensible and justified; however, they do not address whether use of Ologen in any way alters the mechanics of the glaucoma surgery and subsequent IOP management - which again we do not expect. Furthermore, this study describes the use and results of Ologen in one surgeon’s hands. It is possible that results could be different in alternative surgical techniques that may differ in tunnel length and closure.

 As identified by the authors in the discussion, the major limitations of this paper are its small study size and its lack of comparison to other grafts. However it does present a novel method for covering tubes - timely given the shift of glaucoma to a surgical disease. This article serves as a straightforward and succinct study that justifies further investigation and a more robust sub-group analysis.

 Overall, this article is in line with the requirements of F1000Research and we support its approval.

We have read this submission. We believe that we have an appropriate level of expertise to confirm that it is of an acceptable scientific standard.

# Peer review: Referee response for version 1

**DOI:** 10.5256/f1000research.9936.r15375

**Published:** 2016-08-08

**Authors:** Nils Loewen, Yalong Dang

**Affiliations:** 1 Department of Ophthalmology, School of Medicine, University of Pittsburgh, Pittsburgh, PA, USA

Ologen is an FDA approved biodegradable collagen matrix that can be used in trabeculectomy to maintain the bleb space and modulate wound healing ^1^^,^^2^ and repair of scleral defects ^3^. No lyophilized or prepared donor tissue is required which potentially has cost, safety, storage and standardization advantages. The authors are likely some of the most experienced surgeons worldwide with this material and need to be commended for sharing their experience and insight. In this retrospective study, Stephens *et al *evaluated the outcomes of this porcine derived biodegradable collagen matrix as a patch graft in tube shunt surgery. The authors found that 2 eyes out of 43 had tube erosion and required further surgical interventions. Both patients had a history of poor wound healing or ocular inflammation increasing the risk of erosion. It is intriguing that the rate of erosion is lower, 4.7%, compared to other materials or at least similar (indicating non-inferiority).

 This first description of Ologen as a tube patch material is interesting and useful and might standardize tube shunt surgeries while avoiding human donor tissue.

 I believe this already good manuscript can be made more useful by adding the following:

1. Can the authors please report the IOP data before and after surgery as well as number of medications?
2. Power calculation: approximately how many patients would be needed to show that ologen is better than the reported rates for other materials? While not critical it is possible that the patient number is large enough to make this statement and if not, at least non-inferiority is likely. In my opinion, is not a critical issue that should not prevent this article from being approved. 
3. Ologen handling: the authors are extremely experienced with how ologen behaves and a description in the discussion would be helpful for less experienced surgeons. I recall the first generations looked like lifesaver rings but later became less rigid plates or circles?
4. Rosentreter *et al *published 2 papers using Ologen to repair the erosion of tube shunt ^4^  or to modulate wound healing and bleb encapsidation after GDD surgery ^5^. However, in the both studies, Ologen implantation was applied several months after the primary surgery. In the second study, the success rate of the Ologen group was significant lower than the controls (only MMC and capsule excision), indicating this collagen graft might affect the post-surgery intraocular pressure. Could authors please share their thoughts about this in the Discussion? This is mostly likely a result of patient cohort under study (i.e. scar formers).
5. Do the authors use the same postoperative medications with Ologen or can steroids be tapered sooner? Would combining Ologen with a scleral patch make sense in select patients?
6. Could Ologen improve the IOP if placed around the plate to modify encapsidation?
7. What is the authors’ currently prefered practice pattern for tube shunts, scleral patch or Ologen? Or in other words, how do the authors decide when to use Ologen over a scleral patch graft.

We have read this submission. We believe that we have an appropriate level of expertise to confirm that it is of an acceptable scientific standard, however we have significant reservations, as outlined above.

## References

[1] El-SaiedHMAbdelhakimMA: Trabeculectomy with ologen in secondary glaucomas following failed trabeculectomy with MMC: comparative study. *Eye (Lond)*.2016; 10.1038/eye.2016.114 10.1038/eye.2016.114 27256305PMC4985671

[2] CillinoSDi PaceFCillinoGCasuccioA: Biodegradable collagen matrix implant vs mitomycin-C as an adjuvant in trabeculectomy: a 24-month, randomized clinical trial. *Eye (Lond)*.2011;25(12) : 10.1038/eye.2011.219 1598-606 10.1038/eye.2011.219 21921953PMC3234465

[3] ChoCHLeeSB: Biodegradable collagen matrix (Ologen™) implant and conjunctival autograft for scleral necrosis after pterygium excision: two case reports. *BMC Ophthalmol*.2015;15: 10.1186/s12886-015-0130-z 140 10.1186/s12886-015-0130-z 26499993PMC4619331

[4] RosentreterASchildAMDinslageSDietleinTS: Biodegradable implant for tissue repair after glaucoma drainage device surgery. *J Glaucoma*.2012;21(2) : 10.1097/IJG.0b013e3182027ab0 76-8 10.1097/IJG.0b013e3182027ab0 21278584

[5] RosentreterAMelleinACKonenWWDietleinTS: Capsule excision and Ologen implantation for revision after glaucoma drainage device surgery. *Graefes Arch Clin Exp Ophthalmol*.2010;248(9) : 10.1007/s00417-010-1385-y 1319-24 10.1007/s00417-010-1385-y 20405139
